# Characteristics of blood lipid and metabolic indicators in subclinical hypothyroidism patients: a retrospective study

**DOI:** 10.3389/fmed.2024.1439626

**Published:** 2024-10-30

**Authors:** Lili Zhu, Xiaohong Jiang

**Affiliations:** ^1^Chengdu University of Technology Logistics Service Group, Chengdu, China; ^2^Department of Internal Medicine, the Hospital of Chengdu University of Technology, Chengdu, China

**Keywords:** blood lipid, dysglycemia, free tetraiodothyronine, free triiodothyronine, subclinical hypothyroidism, thyroid-stimulating hormone

## Abstract

**Background:**

The characteristics of blood lipid and metabolic indicators were analyzed in patients with subclinical hypothyroidism (SCH), and the effect of type 2 diabetes mellitus (T2DM) on SCH patients was determined.

**Methods:**

The physical examination data of 2,119 residents in a university community were retrospectively divided into 2 groups (SCH and non-SCH groups). Furthermore, the SCH group was divided into SCH-T2DM and SCH–non-T2DM subgroups, and the data between the groups were analyzed.

**Results:**

The SCH group had significantly higher levels of triglycerides (*p* = 0.044), total cholesterol (*p* = 0.001), low-density lipoprotein cholesterol (*p* = 0.019), very low-density lipoprotein cholesterol (*p* = 0.044), 2-h plasma glucose (*p* = 0.023), and globulin (*p* = 0.000) compared to the non-SCH group. The SCH–T2DM group was older (*p* < 0.001), had a greater BMI (*p* = 0.028), a more rapid heart rate (*p* = 0.025), and a greater waist circumference (*p* < 0.001) than the SCH–non-T2DM group based on the subgroup analysis. The SCH–T2DM group had significantly higher dyslipidemia and dysglycemia levels than the SCH–non-T2DM group.

**Conclusion:**

Patients with SCH with or without T2DM may have dyslipidemia and dysglycemia and should be evaluated accordingly.

## 1 Introduction

Subclinical hypothyroidism (SCH) is a medical condition in which thyroid-stimulating hormone (TSH) levels are slightly elevated, while thyroid hormone levels (FT3 and FT4) remain within the normal range (1). SCH correlates with long-term metabolic disorders and is related to congestive heart failure, coronary artery disease events, and fatal stroke (1, 2). SCH is a significant public health concern. The prevalence of SCH is 3–15%, depending on the population (1). The Universal Salt Iodization (USI) program was implemented in China in 1995 and has successfully lowered the prevalence of SCH in 10 cities from 16.7% in 1999 to 3.22% in 2016 (1, 3). The prevalence of SCH in Jiangxi Province, China, was reported to be 7.89% in 2023 (4). SCH is more common in women, the elderly, and individuals with a family history of diabetes and thyroid diseases (1, 5–8). A survey among women in northeast China showed that SCH patients had significantly higher triglyceride (TG) levels (1.69 ± 1.9 vs. 1.45 ± 1.4) than the healthy population (9). Another study by Sindhu and Vijay (10) suggested that SCH patients had more significant dyslipidemia than the euthyroid population, including total cholesterol (TC), very low-density lipoprotein cholesterol (VLDL-C), and low-density lipoprotein cholesterol (LDL-C). Given the potential health consequences of SCH and the correlation with metabolic disorders and other diseases (1–10), there is a need to determine the changes in blood lipid levels and other metabolic indicators in patients with SCH.

Moreover, a positive correlation has been shown between poor glycemic control and a high TSH level in type 2 diabetes mellitus (T2DM) patients and mouse models of T2DM (11). SCH increases insulin resistance in people with normal glucose levels (12). Han et al. (13) reported that T2DM patients are more likely to have SCH than the non-T2DM population, and SCH may be associated with increased diabetic complications and T2DM progression than the non-SCH population. Alharbi et al. (14) showed no association between SCH and T2DM development. Based on the collective findings from these studies, SCH patients with T2DM should receive special attention.

The association between SCH and T2DM development has received wide attention. We have focused our attention on the association between SCH and T2DM in a population group in southwest China. The current study was conducted in southwest China, the purpose of which was to determine blood lipid characteristics and metabolic indicators in patients with SCH in a university community. Moreover, the current study compared SCH patients with or without T2DM to determine the effect of T2DM on SCH patients. This study focused on diverse populations and may be helpful in further understanding SCH patients.

## 2 Patient and methods

### 2.1 Participants

This study was approved by the Ethics Committee of the Hospital of Chengdu University of Technology (No. 2020001). All university community residents who underwent physical examinations at the hospital from March to December 2019 were screened for eligibility.

### 2.2 Diagnostic criteria

Thyroid dysfunction was diagnosed using the standards established by the 2007 *Chinese Guidelines for Diagnosis and Treatment of Thyroid Diseases - Hyperthyroidism* (15) and the 2017 *Guidelines for Diagnosis and Treatment of Adult Hypothyroidism* (16). The usual reference value range for thyroid-related hormones in our institution is as follows: TSH, 0.27–4.0 mIU/ml; FT4, 12–22 pmol/L; and FT3, 3.1–6.8 pmoL/L. The TSH level was elevated, and the FT4 and FT3 levels were normal, which were consistent with SCH.

### 2.3 Data collection

A standard questionnaire was used to collect personal history, past medical history, family history, and other general information from all participants. General clinical indices, including systolic blood pressure (SBP), diastolic blood pressure (DBP), heart rate (HR), body mass index (BMI), waist circumference (W), and hip circumference (H), were measured in the morning. Fasting venous blood was collected, and electrochemiluminescence was used to determine the TSH, FT4, and FT3 levels. Automatic biochemical instruments were used to detect TG, TC, LDL-C, VLDL-C, high-density lipoprotein cholesterol (HDL-C), fasting blood glucose (FPG), urea nitrogen (BUN), creatinine (Scr), uric acid (UA), albumin (A), globulin (G), alanine aminotransferase (ALT), aspartate aminotransferase (AST), alkaline phosphatase (ALP), *γ*-glutamyltransferase (GGT), total bilirubin (STB), conjugated bilirubin (CB), and unconjugated bilirubin (UCB). The postprandial blood glucose (2hPG) level was measured 2 h after a standard steamed bread meal.

### 2.4 Grouping

Based on the findings of thyroid function tests, participants were split into two groups (SCH and non-SCH groups). The non-SCH group had normal thyroid function, and clinical hyperthyroidism, SCH, and hypothyroidism were excluded. The patients with familial dyslipidemia, arterial hypertension, morbid obesity, and nephrotic syndrome, as well as patients taking lipid-lowering treatments (fibrates and HMG-coA inhibitors), were excluded. The clinical baseline data, blood lipid levels, and other metabolic markers in the two groups were compared and examined.

To compare SCH patients with or without T2DM and to determine the effect of T2DM on SCH patients, the SCH patients were divided into SCH–T2DM and SCH–non-T2DM subgroups. The clinical baseline data, blood lipid levels, and other metabolic markers were compared and examined in the two subgroups.

### 2.5 Biochemical parameter assay methods

Biochemical indicators were detected using spectrophotometry (Roche Cobas c 501 automatic biochemical analyzer). TSH, FT4, and FT3 levels were determined using electrochemiluminescence (electrochemical luminescence) assay (Roche Cobas e 411 electrochemiluminescence automatic immunoassay).

### 2.6 Statistical processing

Data processing and statistical analysis were performed using SPSS 25.0. The measurement data were compared across groups using a *t*-test. A statistically significant difference was indicated by a significance level of *p* < 0.05.

## 3 Result

### 3.1 Patients included

Of 3,226 university community residents, 2,119 met the inclusion criteria for this study, including 1,040 males and 1,079 females 23–95 years of age. A total of 1,107 individuals were disqualified and not included in the final analysis. Specifically, 735 patients were excluded because of incomplete physical examination items, and 42 patients had clinical hyperthyroidism, subclinical hyperthyroidism, clinical hypothyroidism, a history of thyroid surgery, a pituitary tumor, or severe parathyroid disease. Additionally, 206 individuals had a history of diabetes, 5 had a recent history of acute and severe infection, 11 had a history of long-term use of glucocorticoids and other drugs that could affect thyroid function, and 12 had a history of malignant tumors. Moreover, 94 individuals had severe heart, liver, and kidney disease, while 3 lacked the ability to comprehend and cooperate with physicians for necessary examinations and treatments.

### 3.2 Comparison of general clinical indices between the SCH and non-SCH groups

The SCH group consisted of 440 patients (20.56%) and the non-SCH group consisted of 1,679 individuals. The SCH group was significantly older than the non-SCH group (*t* = 4.73; *p* < 0.001). The BMI, gender, heart rate, blood pressure, W, and H were not significantly different between the SCH and non-SCH groups (*p* > 0.05). Table 1 provides a detailed presentation of the study results.

**Table 1:** Comparison of general clinical indices between the SCH and non-SCH groups.

	SCH (*n* = 440)	Non-SCH (*n* = 1,679)	*t*	*p*
Age (y)	59.9 ± 16.5	55.6 ± 16.9	4.73	<0.001
Gender (Male/Female)	217/223	842/837	1.86	0.267
BMI (kg/m^2^)	23.4 ± 3.2	23.5 ± 3.1	−0.74	0.458
SBP (mmHg)	126.4 ± 15.7	125.2 ± 15.2	1.46	0.145
DBP (mmHg)	76.9 ± 8.7	77.6 ± 9.3	−1.39	0.164
HR (beat/min)	78.4 ± 10.8	78.4 ± 11.2	0.02	0.982
W(cm)	80.1 ± 9.6	80.5 ± 9.6	−0.78	0.437
H(cm)	95.3 ± 6.4	95.6 ± 6.5	−0.92	0.356

BMI, body mass index; SCH, subclinical hypothyroidism; SBP, systolic blood pressure; DBP, diastolic blood pressure; HR, heart rate; W, waist circumference; H, hip circumference.

### 3.3 Comparison of blood lipid and other biochemical indices between the SCH and non-SCH groups

The TG levels were higher in the SCH group than the non-SCH group (*t* = 2.02; *p* = 0.044), as were the TC (*t* = 3.22; *p* = 0.001), LDL-C (*t* = 2.35; *p* = 0.019), and VLDL-C levels (*t* = 2.01; *p* = 0.044). The SCH group had a lower HDL-C level than the non-SCH group but the difference was not statistically significant (*t* = 0.68; *p* = 0.496). Furthermore, the 2hPG level in the SCH group was significantly higher than in the non-SCH group (*t* = 2.28; *p* = 0.023). The TG level was also significantly higher in the SCH group than in the non-SCH group (*t* = 4.12; *p* < 0.001). No substantial differences were detected in other biochemical indexes between the two groups (*p* > 0.05). Table 2 presents a detailed summary of the results.

**Table 2:** Comparison of blood lipid and other biochemical indices between the SCH and non-SCH groups.

	SCH (*n* = 440)	Non-SCH (*n* = 1,679)	*t*	*p*
TG (mmol/L)	1.58 ± 0.95	1.48 ± 0.94	2.02	0.044
TC (mmol/L)	4.99 ± 1.13	4.81 ± 0.92	3.22	0.001
LDL-C (mmol/L)	2.68 ± 1.02	2.56 ± 0.81	2.35	0.019
VLDL-C (mmol/L)	0.72 ± 0.43	0.67 ± 0.43	2.01	0.044
HDL-C (mmol/L)	1.60 ± 0.46	1.58 ± 0.42	0.68	0.496
FPG (mmol/L)	5.21 ± 0.84	5.16 ± 0.85	1.14	0.254
2hPG (mmol/L)	7.38 ± 2.07	7.11 ± 2.04	2.28	0.023
BUN (mmol/L)	5.23 ± 1.59	5.09 ± 1.51	1.72	0.086
Scr (μmol/L)	75.55 ± 22.15	75.27 ± 24.00	0.22	0.825
UA (μmol/L)	336.61 ± 83.21	337.47 ± 83.95	−0.19	0.848
A (g/L)	46.76 ± 2.58	47.01 ± 2.61	−1.75	0.081
G (g/L)	29.11 ± 3.77	28.23 ± 4.04	4.12	<0.001
AST (u/L)	23.14 ± 11.95	22.36 ± 11.01	1.29	0.196
ALT (u/L)	19.75 ± 13.04	21.06 ± 19.33	−1.35	0.179
ALP (g/L)	77.56 ± 22.13	76.11 ± 22.40	1.21	0.226
GGT (u/L)	30.01 ± 104.14	26.74 ± 29.69	1.13	0.260
STB (μmol/L)	11.83 ± 4.97	12.32 ± 5.07	−1.80	0.072
CB (μmol/L)	3.53 ± 1.33	3.61 ± 1.33	−1.13	0.258
UCB (μmol/L)	8.32 ± 3.79	8.72 ± 3.93	−1.89	0.059

SCH, subclinical hypothyroidism; TG, triglycerides; TC, total cholesterol; LDL-C, low-density lipoprotein cholesterol; VLDL-C, very low-density lipoprotein cholesterol; HDL-C, high-density lipoprotein cholesterol; FPG, fasting blood glucose; 2hPG, 2-h postprandial blood glucose; BUN, urea nitrogen; Scr, creatinine; UA, uric acid; A, albumin; G, globulin; AST, aspartate aminotransferase; ALT, alanine aminotransferase; ALP, alkaline phosphatase; GGT, γ-glutamyltransferase; STB, total bilirubin; CB, conjugated bilirubin; UCB, unconjugated bilirubin.

### 3.4 Comparison of general clinical indices between the SCH–T2DM and SCH–non-T2DM subgroups

For further analysis, the SCH population was categorized into SCH–T2DM and SCH–non-T2DM groups. The SCH–T2DM group was older (*t* = 9.003; *p* < 0.001), the BMI was higher (*t* = 2.203; *p* = 0.028), the heart rate was more rapid (*t* = 2.253; *p* = 0.025), and the W was greater (*t* = 4.142; *p* < 0.001) than the SCH–non-T2DM group. The other indices we measured were not significantly different between the two groups (*p* > 0.05). Table 3 provides a detailed presentation of the study results.

**Table 3:** Comparison of general clinical indices between the SCH–T2DM and SCH–non-T2DM groups.

	SCH–T2DM (*n* = 60)	SCH–non-T2DM (*n* = 380)	*t*	*p*
Age (y)	75.50 ± 10.95	59.86 ± 16.49	9.003	<0.001
BMI (kg/m^2^)	24.45 ± 2.86	23.42 ± 3.16	2.203	0.028
HR (beat/min)	82.06 ± 11.05	78.43 ± 10.78	2.253	0.025
SBP (mmHg)	138.18 ± 15.56	126.37 ± 15.68	5.051	<0.001
DBP (mmHg)	77.78 ± 9.89	76.89 ± 8.74	0.672	0.502
W (cm)	85.90 ± 8.34	80.05 ± 9.57	4.142	<0.001
H (cm)	95.88 ± 6.21	95.26 ± 6.38	0.655	0.512

BMI, body mass index; SCH, subclinical hypothyroidism; SBP, systolic blood pressure; DBP, diastolic blood pressure; HR, heart rate; W, waist circumference; H, hip circumference.

### 3.5 Comparison of blood lipid and other biochemical indices between the SCH–T2DM and SCH–non-T2DM groups

Table 4 shows the comparisons of blood lipid and other biochemical indices between the SCH–T2DM and SCH–non-T2DM groups. The TG (*t* = 2.159; *p* = 0.036), TC (*t* = −2.147; *p* < 0.001), LDL-C (*t* = −2.541; *p* = 0.011), and VLDL-C levels (*t* = 2.158; *p* = 0.036) were significantly higher in the SCH–T2DM group than the SCH–non-T2DM group. The FPG (*t* = 8.886; *p* < 0.001), 2hPG (*t* = 6.725; *p* < 0.001), BUN (*t* = 3.236; *p* = 0.001), Scr (*t* = 2.170; *p* = 0.031), UA (*t* = 2.775; *p* = 0.006), A (*t* = −2.711; *p* = 0.007), and G levels (*t* = 3.696; *p* < 0.001) were also significantly different between the two groups.

**Table 4:** Comparison of blood lipid and other biochemical indices between the SCH–T2DM and SCH–non-T2DM groups.

	SCH–T2DM (*n* = 60)	SCH–non-T2DM (*n* = 380)	*t*	*p*
TG (mmol/L)	2.22 ± 2.06	1.58 ± 0.95	2.159	0.036
TC (mmol/L)	4.63 ± 1.22	4.99 ± 1.13	−2.147	<0.001
LDL-C (mmol/L)	2.29 ± 1.00	2.68 ± 1.02	−2.541	0.011
VLDL-C (mmol/L)	1.01 ± 0.94	0.72 ± 0.43	2.158	0.036
HDL-C (mmol/L)	1.34 ± 0.38	1.60 ± 0.46	−3.799	<0.001
FPG (mmol/L)	8.08 ± 2.26	5.21 ± 0.84	8.886	<0.001
2hPG (mmol/L)	11.66 ± 4.07	7.38 ± 2.07	6.725	<0.001
BUN (mmol/L)	6.04 ± 2.31	5.23 ± 1.59	3.236	0.001
Scr (μmol/L)	82.94 ± 28.21	75.55 ± 22.15	2.170	0.031
UA (μmol/L)	371.74 ± 98.04	336.61 ± 83.21	2.775	0.006
A (g/L)	45.70 ± 3.04	46.76 ± 2.58	−2.711	0.007
G (g/L)	31.22 ± 4.46	29.11 ± 3.77	3.690	<0.001
AST (u/L)	21.48 ± 4.74	23.14 ± 11.95	−0.973	0.331
ALT (u/L)	20.42 ± 8.46	19.75 ± 13.04	0.355	0.723
ALP (g/L)	81.32 ± 22.02	77.56 ± 22.13	1.138	0.255
GGT (u/L)	26.96 ± 17.54	30.01 ± 14.14	−0.207	0.836
STB (μmol/L)	11.01 ± 3.81	11.83 ± 4.97	−1.131	0.258
CB (μmol/L)	3.58 ± 1.25	3.53 ± 1.33	0.284	0.776
UCB (μmol/L)	7.43 ± 2.74	8.32 ± 3.79	−1.620	0.106

SCH, subclinical hypothyroidism; TG, triglycerides; TC, total cholesterol; LDL-C, low-density lipoprotein cholesterol; VLDL-C, very low-density lipoprotein-cholesterol; HDL-C, high-density lipoprotein cholesterol; FPG, fasting blood glucose; 2hPG, 2-h postprandial blood glucose; BUN, urea nitrogen; Scr, creatinine; UA, uric acid; A, albumin; G, globulin; AST, aspartate aminotransferase; ALT, alanine aminotransferase; ALP, alkaline phosphatase; GGT, γ-glutamyltransferase; STB, total bilirubin; CB, conjugated bilirubin; UCB, unconjugated bilirubin.

## 4 Discussion

SCH is the most prevalent type of thyroid dysfunction, as confirmed by numerous epidemiologic studies. SCH may be affected by geographic location, ethnicity, dietary patterns (e.g., iodine intake), age, and gender (1–10, 17). A community-based investigation in China showed that the population with SCH was older (17), which is consistent with the results of our study. This finding may be attributed to thyroid gland atrophy and fibrosis, reduced synthesis and release of thyroid hormones, and decreased levels of follicular epithelial cells, glia, and secretory granules in the follicles, especially among elderly individuals. Additionally, autoimmunity changes and a relative iodine deficiency in the elderly may also contribute to the decline in thyroid function (18). SCH usually affects the female population more than the male population (1–8). The number of males and females in the SCH group did not differ significantly, which may be due to the retrospective nature of the study based on a single-center community.

Numerous studies have shown that SCH can lead to dyslipidemia, which is characterized by decreased levels of HDL-C and elevated levels of TG, TC, and LDL-C (19, 20). While the levels of FT3 and FT4 are negatively correlated with TG and HDL-C and positively correlated with TC and LDL-C, respectively, TSH is favorably correlated with TG and TC and negatively correlated with HDL-C (19, 20).

Inconsistent findings have been reported with respect to the impact of SCH on the blood lipid profile. Luo et al. (21) reported no conclusive correlation between SCH and TC, LDL-C, or HDL-C among >11,000 patients undergoing physical examinations. In a study involving 11,498 participants, Nakajima et al. (22) concluded that SCH was correlated with higher W, TG, and LDL-C levels in females. An increase in TC levels in females is likewise linked to the progression from euthyroidism to SCH (22). Khan et al. (23) showed that LDL-C, non-HDL-C, UA, and the albumin-to-creatinine ratio increased from euthyroidism to overt hypothyroidism, with more subtle changes observed in SCH. A meta-analysis revealed that individuals with SCH had notably higher levels of TC, TG, and LDL-C, but no significant difference was detected in the HDL-C level compared to individuals with normal thyroid function (24). Similarly, the findings in the current study showed that individuals with SCH had TG, TC, LDL-C, and VLDL-C levels that were considerably higher than among individuals in the non-SCH group. The difference was not statistically significant even though the HDL-C level was lower than the non-SCH group.

SCH is closely associated with impaired glucose metabolism. The prevalence of SCH in the T2DM group was 16.39% compared to 11.27% in the normoglycemic group (12). The SCH–T2DM patients had poor blood glucose control (25, 26), and there was a bidirectional relationship between the SCH and T2DM. The FBG, 2hPG, and HbA1c levels were significantly lower in hypothyroid patients than in healthy individuals in China (27). However, another study (28) showed that patients with SCH and T2DM had higher blood glucose levels than patients without hypothyroidism with relatively lower fasting blood glucose levels and a more significant difference in the 2-h postprandial blood glucose level. There were no appreciable differences between the population with SCH and the non-SCH group in the current study with respect to other metabolic markers. These metabolic indicators included fasting blood glucose, liver function, renal function, blood UA, SBP, DBP, heart rate, BMI, W, and H. The SCH patients combined with T2DM had significantly higher levels of dyslipidemia and dysglycemia than patients without T2DM, which is consistent with previous studies (27, 28). This finding indicates that SCH–T2DM patients may be at risk for severe complications and more obvious clinical symptoms. Therefore, healthcare providers should be vigilant and consider SCH as a potential diagnosis in T2DM patients.

Autoimmunity is considered the main etiology of SCH. Numerous studies have reported a high positivity rate of thyroid autoantibodies in patients with SCH (29, 30). Shi et al. (29) reported that 33.1% of SCH patients have autoimmune thyroid disease, with higher rates of thyroid peroxidase antibody and thyroglobulin antibody than the general population. Although no specific thyroid-related antibodies were detected in the current study, the SCH group exhibited a higher G level than the non-SCH group, which could be attributed to the increased presence of autoantibodies.

Yang et al. (12) reported significant differences in the BMI, HOMA-IR, and waist-to-hip ratio in the SCH–T2DM group compared to the SCH–non-T2DM group. This study also showed older age, a higher BMI, and a thicker W in the SCH–T2DM group than in the SCH–non-T2DM group. These results were similar to the study by Yang et al. (12).

In the future, it will be important to continue researching the mechanisms underlying the metabolic disorders associated with SCH to develop effective interventions and treatments. In addition, more research is needed to investigate the link between SCH and cardiovascular risk, as well as other potential health implications. The need to educate healthcare professionals and the general public about the dangers of SCH and the need for routine screening and monitoring is critical given the high incidence. Furthermore, because SCH is often asymptomatic, future research should focus on identifying biomarkers or other indicators that can help to identify individuals who are at risk of developing this condition and on developing targeted prevention strategies. Finally, the possible contribution of lifestyle variables, including food and exercise, to the therapy of metabolic diseases linked to SCH warrants further investigation.

This study was limited for the following reasons: First, this was a single-center community retrospective analysis. Second, confounding factors such as cigarette smoking, alcohol consumption, and physical activity, which may influence SCH and T2DM, were not analyzed. Third, this study only included the residents of a university community in southwest China. This population’s age, ethnicity, and education may have biased the results.

## 5 Conclusion

SCH patients in this community were shown to exhibit metabolic disorders (LDL-C, VLDL-C, TG, and elevated postprandial blood glucose). SCH patients with T2DM had significantly higher levels of dyslipidemia and dysglycemia than SCH patients without T2DM. Regular monitoring of this population’s blood lipid and glucose levels is advised for early intervention and prevention of complications.

## Data Availability

The original contributions presented in the study are included in the article/supplementary material, further inquiries can be directed to the corresponding author.
